# Evaluation of Clinical Outcomes and Simultaneous Digital Tracking of Daily Physical Activity, Heart Rate, and Inhalation Behavior in Patients With Pulmonary Arterial Hypertension Treated With Inhaled Iloprost: Protocol for the Observational VENTASTEP Study

**DOI:** 10.2196/12144

**Published:** 2019-04-15

**Authors:** Christian Mueller, Barbara Stollfuss, Alexander Roitenberg, Jonas Harder, Manuel J Richter

**Affiliations:** 1 Bayer Vital GmbH Leverkusen Germany; 2 xbird GmbH Berlin Germany; 3 Department of Internal Medicine Justus-Liebig-University Giessen Universities of Giessen and Marburg Lung Center, Member of the German Center for Lung Research (DZL) Giessen Germany

**Keywords:** digital monitoring, heart rate, daily physical activity, inhalation, behavior, pulmonary arterial hypertension, iloprost, Breelib, health-related quality of life, sleep

## Abstract

**Background:**

Pulmonary arterial hypertension (PAH)—a progressive, ultimately fatal disease—patients often experience dyspnea, which can limit their daily physical activities. Iloprost is an inhaled therapy for PAH that has shown efficacy in clinical trials. However, clinical trials in PAH have provided only limited data on daily physical activity. Digital monitoring of daily physical activity in PAH is therefore attracting growing interest. To fully understand a patient’s response to treatment, monitoring of treatment adherence is also required. The Breelib nebulizer for administration of iloprost saves inhalation data, thus allowing digital monitoring of adherence.

**Objective:**

This study aims to perform parallel digital tracking of daily physical activity parameters, heart rate, and iloprost inhalation data in patients with PAH, before and after starting inhaled iloprost treatment. The primary objective is to investigate correlations between changes in digital measures of daily physical activity and traditional clinical measures. Secondary objectives are to assess iloprost inhalation behavior, the association between daily physical activity measures and time since last inhalation, changes in sleep quality and heart rate, the association of heart rate with daily physical activity measures and iloprost inhalation, and adverse events.

**Methods:**

VENTASTEP is a digital, prospective, observational, multicenter, single-arm cohort study of adults with PAH in Germany, starting inhaled iloprost treatment via the Breelib nebulizer, in addition to existing PAH therapy. The study comprises a baseline period without iloprost treatment (≤2 weeks) and an observation period with iloprost treatment (3 months±2 weeks). The Apple Watch Series 2 and iPhone 6s are used with a dedicated study app to continuously measure digital daily physical activity parameters and heart rate during the baseline and observation periods; the watch is also used with a 6-min walk distance (6MWD) app to measure digital 6MWD at baseline and the end-of-observation visit. Inhalation frequency, completeness, and duration are monitored digitally via the nebulizer and the BreeConnect app. Sleep quality is assessed using the Pittsburgh Sleep Quality Index at baseline and the end-of-observation visit. Changes in traditional outcome measures (6MWD, Borg dyspnea scale, EuroQol 5-dimensions questionnaire, functional class, and brain natriuretic peptide [BNP] or N-terminal proBNP) between baseline and the end-of-observation visit will be correlated with changes in digital daily physical activity parameters and digital 6MWD as the primary analysis.

**Results:**

The first participant was enrolled in February 2018 (estimated study completion by July 2019; planned sample size: 80 patients).

**Conclusions:**

The VENTASTEP study will inform future research on the utility of digital parameters as outcome assessment tools for disease monitoring in PAH. The study will also provide insight into clinical outcomes, daily physical activity, and quality of life in patients adding inhaled iloprost, to existing PAH therapy.

**Trial Registration:**

ClinicalTrials.gov NCT03293407; https://clinicaltrials.gov/ct2/show/NCT03293407 (Archived by WebCite at http://www.webcitation.org/6ywPGcn4I)

**International Registered Report Identifier (IRRID):**

DERR1-10.2196/12144

## Introduction

### Background

Pulmonary arterial hypertension (PAH) is a progressive disease in which increased pulmonary vascular resistance leads to right heart failure and death. The most common symptom of PAH is persistent dyspnea on exertion, which can limit the ability of patients to perform ordinary daily physical activities [1].

Iloprost is an inhaled prostacyclin-based therapy for PAH that reduces pulmonary vascular resistance through vasodilatory and antiproliferative effects [2-4]. Inhaled iloprost was shown to have a rapid onset of action and improved 6-min walk distance (6MWD), pulmonary vascular resistance, symptoms, and health-related quality of life (HRQoL) at 12 weeks compared with placebo in patients with PAH or chronic thromboembolic pulmonary hypertension in a phase 3 randomized controlled trial [2]. Iloprost also showed beneficial effects when added to the endothelin receptor antagonist (ERA) bosentan [3] and when added to dual combination therapy with ERAs and phosphodiesterase-5 inhibitors [5] in patients with PAH. According to current treatment recommendations, iloprost may be added to dual combination therapy to ensure timely treatment escalation for patients with intermediate risk who respond inadequately to initial therapy [6].

Clinical trials of inhaled iloprost and other pharmacotherapies in PAH have thus far provided only limited data on parameters such as daily physical activity that directly measure the impact of the disease on daily life [7]. Daily physical activity can now be measured continuously with wearable technology that is available to consumers and widely used, providing unprecedented opportunities for biomedical research [8]. Digital monitoring of daily physical activity is therefore attracting growing interest as a potential outcome measure in PAH [9-11], with observational studies showing reduced daily physical activity in patients with PAH compared with controls without PAH [11-13] and significant associations between digital daily physical activity parameters and maximal inspiratory and expiratory pressures [14], right ventricular and pulmonary vascular status [15], peripheral muscle oxygenation [15], 6MWD [9-12], HRQoL [9,10,15,16], and transplantation-free survival [16]. These observational data suggest that digital monitoring of daily physical activity has the potential to become an important tool in the evaluation of disease course and treatment response in PAH. Currently, the 6MWD is used to guide treatment decisions in PAH [17,18] but is assessed only intermittently (eg, every 12 weeks), leaving long intervals in which physicians have no information on the activity status of their patients. Continuous measurement of daily physical activity (eg, as part of a patient support program) could allow earlier detection of deterioration and more rapid adjustment of PAH treatment.

The emerging potential of digital approaches for monitoring and improving treatment adherence and linking adherence to physiological measures was recently highlighted in patients with respiratory diseases [19]. Daily physical activity in patients with PAH has not yet been assessed with simultaneous digital monitoring of adherence to PAH treatment. The recently approved Breelib nebulizer (Vectura Group plc) for administration of iloprost saves inhalation data, thus allowing digital monitoring of adherence [20,21].

### Objectives

This study aims to perform parallel digital tracking of daily physical activity parameters, heart rate, and iloprost inhalation data in patients with PAH, before and after starting treatment with inhaled iloprost. The primary objective is to investigate the correlation between changes in digital daily physical activity measures and changes in traditional clinical measures of disease course and treatment response. Secondary objectives are to assess iloprost inhalation behavior, the association between daily physical activity measures and time since last inhalation, changes from baseline in sleep quality and heart rate (at rest and during 6MWD test), the association of heart rate with daily physical activity measures and iloprost inhalation, and adverse events. Other objectives are to assess activity status (active, inactive, and watch not worn), assess the feasibility of digital measurement of 6MWD by comparing digital and traditional 6MWD measurements, identify digital measures linked to outcomes of special interest such as 6MWD, and evaluate changes from baseline in clinical outcome measures, daily physical activity, and HRQoL that occur when inhaled iloprost is added to existing PAH therapy.

## Methods

### Study Design

VENTASTEP is a digital, prospective, observational, multicenter, single-arm cohort study of adult patients with PAH in Germany, starting treatment with inhaled iloprost via the Breelib nebulizer in a real-world setting.

Heart rate and daily physical activity parameters are monitored digitally using a wearable and a smartphone (the Apple Watch Series 2, 42 mm, Apple Inc and iPhone 6s, Apple Inc; supplied by Vodafone GmbH; see Discussion section for rationale) with a dedicated study app and a 6MWD app (both created by xbird GmbH). Inhalation data (including the average number of daily inhalations, the average daily proportion of complete and incomplete inhalations, and average daily inhalation duration per session) are monitored digitally via the nebulizer and the BreeConnect app (Bayer AG). Traditional clinical outcome measures (eg, physical examination, 6MWD, and laboratory values) are assessed by the investigators, and sleep quality and HRQoL are assessed as patient-reported outcomes.

Patients can only be enrolled in the study if the decision to treat with iloprost has been made by the treating physician in advance and independently of study inclusion. Patients routinely treated within specialized PAH centers and meeting the criteria for enrollment are asked to participate in the study by their physician. Enrolled patients are informed by their investigator about the study objectives and the digital methods applied.

Furthermore, study participants are trained in the correct handling of the wearable and smartphone. Training in the use of the nebulizer is conducted in accordance with routine procedures on behalf of the Ventavis patient support program VENTAPLUS (implemented by Contra Care GmbH, Nürnberg, Germany).

Final data analysis will be performed by the contract research organization (CRO) Institut Dr. Schauerte, Munich, Germany.

### Ethical Considerations

The study protocol has been approved by the ethics committee of the Justus Liebig University Giessen (approval no. AZ 153/17). Before documentation of any data, informed consent is obtained from the patient in writing.

### Patients

Patients with PAH (pulmonary hypertension group 1 according to the current clinical classification [18]) at intermediate risk and in World Health Organization functional class (WHO FC) III are eligible for inclusion in the study, if they have shown an inadequate response to initial therapy with one or more PAH drugs or clinical deterioration after an initial treatment response, and a therapy escalation with inhaled iloprost (administered via the Breelib nebulizer) has been agreed by the patient and physician independently of the study. Other inclusion criteria are patients aged 18 years or above at the initiation of inhaled iloprost, no previous treatment with inhaled iloprost, willingness to wear the Apple Watch Series 2 for the duration of the study, and signed informed consent. Patients are excluded if they are allergic to nickel and methacrylates (which are present in the Apple Watch Series 2) or if they are participating in an investigational program with interventions outside of routine clinical practice.

### Study Devices

#### Apple Watch Series 2

The Apple Watch Series 2 is a wrist-worn, commercially available wearable with a 3-axis gyroscope, a 3-axis accelerometer, Bluetooth 4.0, and optical (photoplethysmography) heart rate sensors. A built-in global positioning system (GPS) sensor enables more accurate distance and pace measuring. GPS data are not captured continuously in this study; only significant GPS location changes (≥500 m) are recorded.

#### iPhone 6s

The iPhone 6s is a commercially available smartphone. Its functionality has been reduced to the following minimum requirements for this study: measurement of movement (using the built-in 3-axis gyroscope and 3-axis accelerometer), measurement of atmospheric pressure (using the built-in barometer), and gathering of data from the nebulizer and the wearable. For data that are gathered by both the wearable and the smartphone (eg, accelerometer data), the Apple iOS automatically selects the more reliable data source for each second of usage.

#### Breelib Nebulizer

The Breelib device is a handheld, battery-powered, vibrating mesh nebulizer for administration of inhaled iloprost that automatically saves inhalation data such as frequency, completeness, and duration of inhalations. The data are transferred to a smartphone using Bluetooth and are stored in the BreeConnect app. A summary of the data can be sent to the treating physician and/or nurse at regular intervals.

### Apps

The study app (developed by xbird) and BreeConnect app (developed by Bayer) are preinstalled on the smartphones used in this study; the 6MWD app (developed by xbird) is preinstalled on the wearables. Once installed on a specific smartphone, the study app automatically creates a numeric unique identifier, which is used as a pseudonym for all data related to that device.

The study app automatically captures daily physical activity and heart rate data from the built-in sensors of the wearable and the smartphone for the duration of the study period. Some of the sensor data are preprocessed by the operating system (Apple Health Kit, Core Location, and Core Motion) before being captured by the study app. All data passively collected by the study app are listed in Table 1.

The 6MWD app saves the number of steps taken, heart rate, and distance walked during the 6MWD tests. The BreeConnect app allows visualization and analysis of inhalation data recorded by the Breelib nebulizer and can also remind the patients when their next iloprost inhalation session is due, if this option is activated by the patients.

**Table 1:** Data captured by the smartphone and wearable and saved in the study app.

Source	Data saved in study app
Health Kit (only access to the listed data is preset in the study app)	Number of steps^a^
	Number of stairs
	Walking distance, m^a^
	Mean heart rate, beats per min^a^
	Number of standing up events
Core Motion (preprocessed data from motion sensors)	Walking time, s
	Stationary time, s
	Exercise time (physically active, more than walking), s
	Automotive time, s
	Cycling time, s
Core Location	Relevant position changes, leaving home location (no continuous data recording; position accuracy: 500 m)^b^
Accelerometer and gyroscope raw data	Heart rate during activity, beats per minute

^a^Steps, walking distance, and mean heart rate per min are also saved in the 6-min walk distance app.

^b^Leaving home location means leaving the 500 m circle that is flagged as *home*. Daily physical activities over a distance <500 m are detected through steps and raw accelerometer and barometer data.

The study app and 6MWD app stay in the background and do not interact with the patient or the investigator, present any obtained physiological values or results, or give recommendations. These 2 apps are neither medical nor lifestyle products as they serve only as vehicles for scientific data collection.

### Data Transfer and Processing

**Figure 1:**
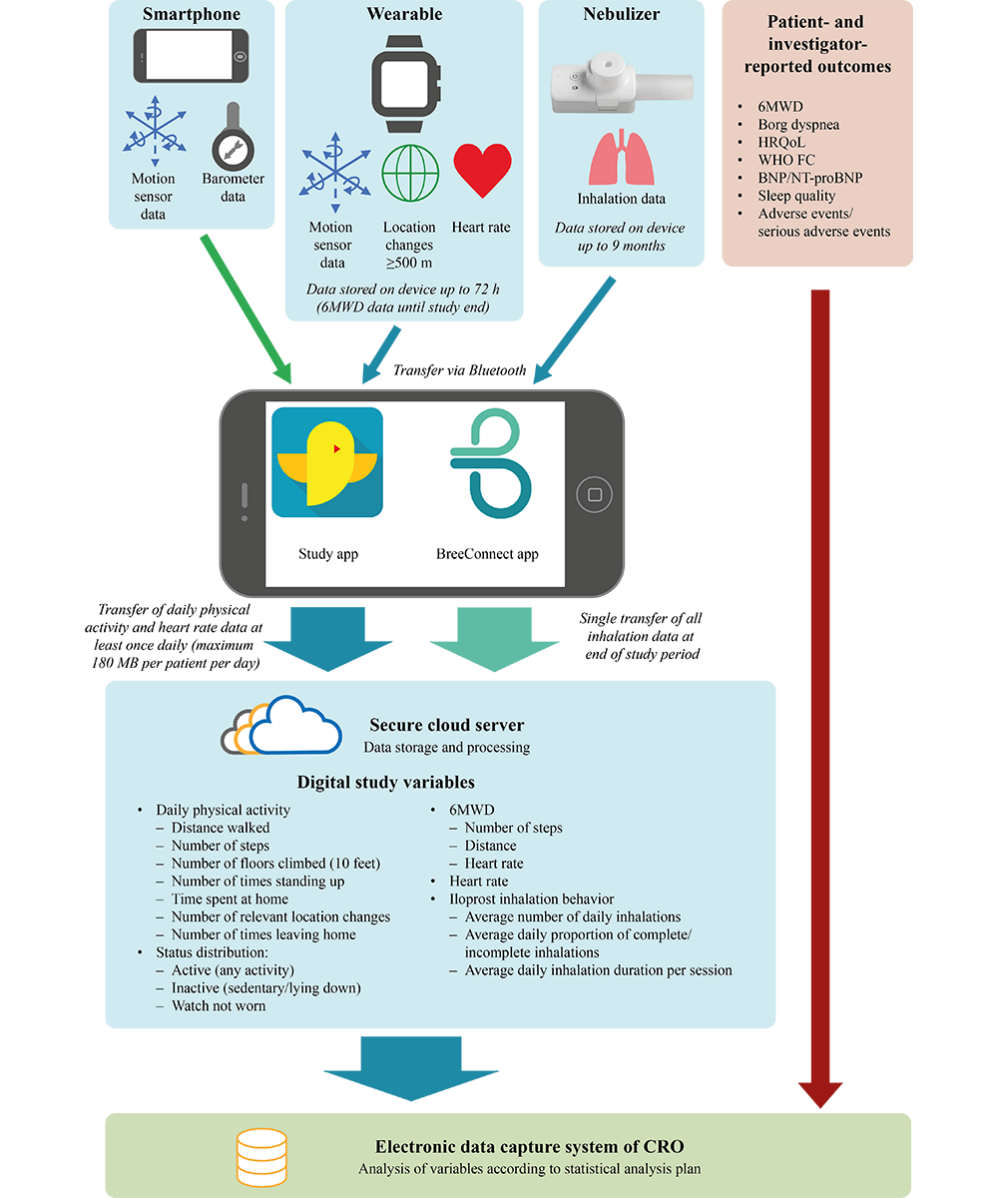
Data sources and processing in the VENTASTEP study. Inhalation data from the nebulizer and motion sensor data, location changes ≥500 m, and heart rate data from the wearable are transferred to the smartphone via Bluetooth and stored there temporarily by the BreeConnect app and study app. Motion sensor and barometer data from the smartphone itself are also stored in the study app. The data are sent from the smartphone via encrypted transmission to a secure cloud server for storage and processing to generate the digital study variables, which are then transferred to the electronic data capture system of the clinical research organization for final analysis. Patient- and investigator-reported outcomes are also saved and analyzed in the electronic data capture system of the clinical research organization. 6MWD: 6-min walk distance; BNP: brain natriuretic peptide; CRO: clinical research organization; NT-proBNP: N-terminal pro–brain natriuretic peptide; HRQoL: health-related quality of life; WHO FC: World Health Organization functional class.

Daily physical activity and heart rate data are sent by Bluetooth from the wearable to the study app on the smartphone (Figure 1). Sensor data from the smartphone itself are also stored by the study app. The daily physical activity and heart rate data on the smartphone are retrieved automatically at least once a day and stored pseudonymized on a secure cloud server.

Inhalation data are sent from the nebulizer to the BreeConnect app on the smartphone by a VENTAPLUS nurse (Contra Care GmbH) during a routine visit with the patient after 3 months of treatment. The data are then automatically transferred to the secure cloud server (Figure 1).

Within the cloud server, the data are processed and formatted into the required study variables (Figure 1). At the individual end of the study for each patient, the processed data are transferred from the cloud server via encrypted connection to the electronic data capture system of the CRO for final analysis. Further information is available in Multimedia Appendix 1.

### Data Collection and Outcome Measures

**Figure 2:**
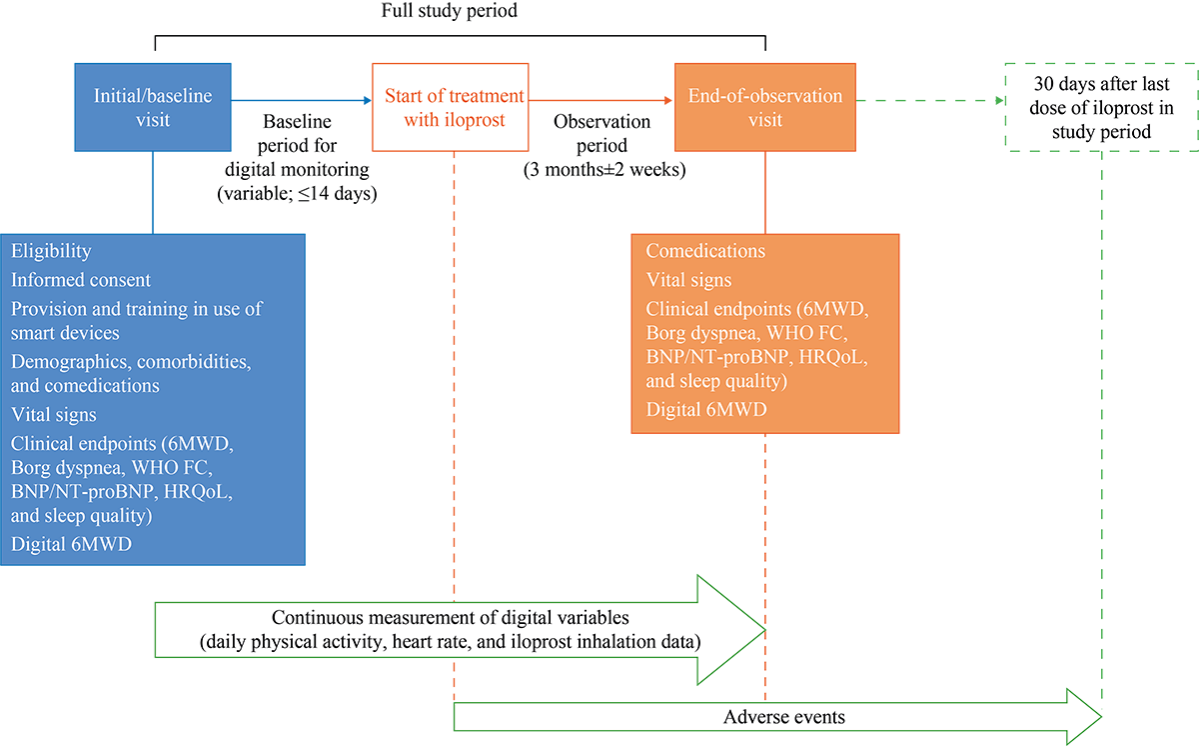
Study visits and data collection. 6MWD: 6-min walk distance; BNP: brain natriuretic peptide; NT-proBNP: N-terminal pro–brain natriuretic peptide; HRQoL: health-related quality of life; WHO FC: World Health Organization functional class.

The whole study period for each patient comprises a baseline period and an observation period (Figure 2). The baseline period is defined as the period from the initial visit and decision to use inhaled iloprost until the start of treatment with inhaled iloprost, or the last 14 days before the start of treatment, if the period from the initial visit to the start of treatment is more than 14 days. This period is variable depending on the time needed to obtain the Breelib nebulizer, fill a prescription, and schedule a visit to train the patient in the use of the device. The observation period is defined as 3 months±2 weeks starting from the first administration of inhaled iloprost.

After signed informed consent has been given, traditional clinical and patient-reported outcomes and digital 6MWD data are collected during the initial routine clinical visit and at 3 months±2 weeks (Figure 2). HRQoL is assessed using the EuroQol 5-dimensions (EQ-5D) questionnaire, and sleep quality is assessed using the Pittsburgh Sleep Quality Index. The investigator collects demographic data and clinical characteristics from the medical records, if available, or else by interviewing the patient. Similarly, the investigator collects effectiveness and safety-related data at the initial and end-of-observation visits. Daily physical activity and heart rate are monitored continuously, and iloprost inhalation data are recorded throughout the whole study (ie, the baseline and observation periods). Resting heart rate is calculated by the Apple system as the average heart rate measured at times of inactivity (on the basis of accelerometer data).

### Statistical Analyses

Statistical analyses of primary and secondary endpoints will be exploratory and descriptive. This study is not designed to confirm or reject predefined hypotheses. Patients receiving at least one dose of inhaled iloprost will be included in the analyses.

#### Primary Analysis

In total, 13 parameters are included in the primary analysis (5 routine clinical parameters: 6MWD, Borg dyspnea scale, HRQoL, WHO FC, and BNP or NT-proBNP, and 8 digital parameters: 6MWD and 7 parameters reflecting daily physical activity, as listed in Figure 1). Digital parameters reflecting daily physical activity will be summarized at baseline (median of all up to 14 device-based daily assessments before the first intake of inhaled iloprost) and during the last 14 days of the observation period (median of daily assessments). If there are no data before the first intake of inhaled iloprost or in the last 14 days of the observation period, the patient will be excluded from the primary analysis.

Patient-wise data (absolute values at baseline and the end of the observation period and changes from baseline to the end of the observation period) will be analyzed descriptively by sample statistics (ie, mean, SD, median, quartiles, and minimum and maximum values). A 95% CI of the mean will also be derived (missing values will be neglected).

For all 78 combinations of the 13 parameters included in the primary analysis, changes from baseline to the end of the observation period will be compared in a correlation analysis, and Pearson and Spearman correlation coefficients will be calculated. Each correlation analysis will be based on pairwise complete observations, with no imputation of missing values.

#### Other Analyses

An exploratory *t* test (2-sided alpha=.05) will be performed to evaluate the statistical significance of changes in 6MWD from the baseline to the end of the observation period. Correlation of step frequency and heart rate measured digitally during the 6MWD test will be assessed.

Regression analyses will be performed for each of the 5 primary clinical endpoints, with a change in the clinical endpoint as a dependent variable and digital endpoints as independent variables. Sensitivity analyses will include correlations of primary endpoints using the full data set rather than pairwise complete observations. The impact of nebulizing time will also be evaluated.

#### Patient Population Size

On the basis of results of a feasibility study conducted in 16 German sites in Q1 2017 (data not shown), the planned total sample size for this exploratory study is 80 patients (minimum value of n=50; maximum value of n=100), who are to be enrolled at about 15 sites over a 12-month period. If fewer than 50 patients are enrolled in 12 months, the recruitment period will be prolonged until 50 patients are enrolled. The minimum number of patients was determined as 50 to ensure reasonable precision of the correlation coefficients (even with 40% missing data). The maximum number of patients was determined as 100 because of financial and organizational reasons.

## Results

### Feasibility Survey

The feasibility of performing a digital noninterventional study was evaluated in an anonymous survey of patients with PAH at a single center (Giessen) in 2017. The patients were asked whether or not they would participate in a hypothetical digital noninterventional study, and under which preconditions.

In total, 30 patients completed the survey questionnaire. However, 15 of the patients indicated that they would not participate in such a study. The reasons for nonparticipation included the following: “too big hurdle”, “too exhausting”, “too much monitoring”, “doubts about guaranteeing patient safety and privacy”, “sitting in a wheelchair”, and “feeling too old”.

The remaining 15 patients indicated that they would participate and use digital devices such as an Apple watch and an iPhone. Of this subset, 8 patients would allow digital monitoring of all variables proposed in the questionnaire (sleep behavior, number of steps, distance traveled, number of visited places, type of movement, GPS location, heart rate, and number/time of meals per day). The other 7 patients would allow digital monitoring of subsets of the proposed variables (measurement of number of steps, distance traveled, and heart frequency were each allowed by 5 of these patients, and measurement of sleep behavior and type of movement were each allowed by 4 of these patients).

### Consequences for Study Protocol

The survey responses suggested that digital monitoring of sleep behavior and energy expenditure and precise GPS tracking would not be accepted by patients; the VENTASTEP protocol therefore excluded digital monitoring of these parameters and included only rough location tracking (changes of ≥500 m). The first participant in the VENTASTEP study was enrolled on February 1, 2018, and the estimated study completion date is July 31, 2019.

## Discussion

### Rationale for Study Design

The VENTASTEP study will evaluate the correlation between changes in a range of digital daily physical activity parameters and changes in established clinical measures of disease course and treatment response in patients with PAH receiving treatment with inhaled iloprost. In addition, the study will provide information on iloprost inhalation behavior and relationship with daily physical activity, the association of heart rate with daily physical activity and iloprost inhalation, adverse events, the feasibility of digital assessment of 6MWD, and changes in clinical outcome measures, sleep quality, heart rate, daily physical activity, and HRQoL that occur when inhaled iloprost is added to existing PAH therapy.

Daily physical activity can be measured using a variety of methods. Self-reporting (eg, via a diary or questionnaire) is inexpensive but subjective [22]. Video recording may be useful in a laboratory setting [23] but is unlikely to be practical for monitoring of daily physical activity in real life [22]. Mechanical pedometers are the simplest wearable daily physical activity sensors, but they provide only step counts without information on exercise intensity [22]. Accelerometers and gyroscopes provide information on linear acceleration and angular motion, respectively, which can be classified into activity types such as walking [22]. Accelerometers have undergone remarkable advances in recent years, with improvements in memory and battery capacities, acceleration range, and linearity, and reductions in size and cost. Furthermore, raw data are being made available to researchers in addition to processed, manufacturer-specific *count* data [24].

We chose to use the Apple Watch Series 2 and iPhone 6s for this study as they allow measurement of many different parameters (they are both equipped with accelerometers and gyroscopes, the watch has a GPS sensor and heart rate monitor, and the smartphone has a barometer). The watch is also simple to wear and relatively unobtrusive, which is an important consideration for acceptability in long-term use. The ActiGraph has been used in several studies in PAH but requires a chest strap to monitor heart rate (similar to some other activity trackers used in medical research), which would not be feasible for a continuous 3-month study. Consumer wearables, including Apple Watches, have been shown to provide reliable measurements of heart rate, number of steps, and distance walked [23,25,26], and the Apple Watch Series 2 was considered the most accurate available consumer wearable at the time of design of the VENTASTEP study (a recent study of 4 wrist wearables in 50 healthy adults showed that the Apple Watch was the most accurate for heart rate monitoring [27], and the ability of the Apple Watch to detect pulse irregularity is now being assessed in 419,093 volunteers in the Apple Heart Study [28]). However, it should be noted that consumer activity trackers tend to have better test-retest reliability and validity for step-counting at average and vigorous walking speed than at slow walking speed [29], which may be relevant for studies in PAH.

**Figure 3:**
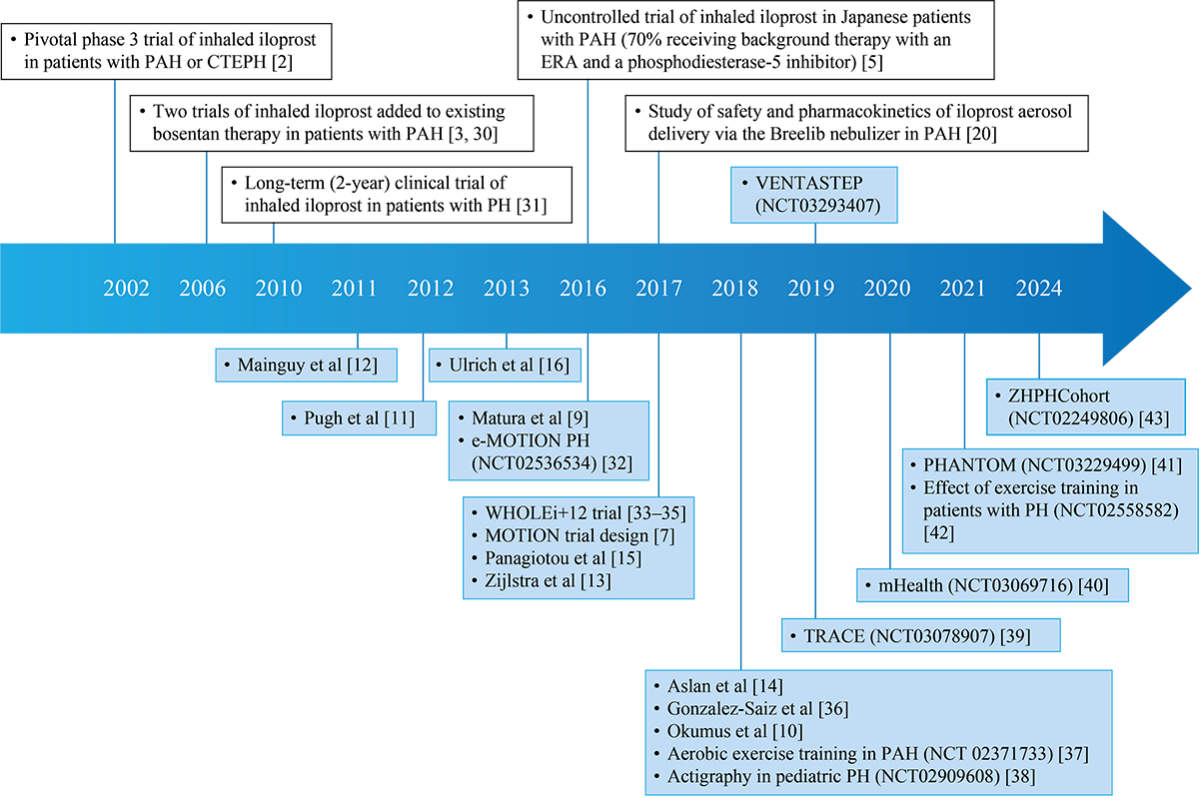
Timeline of key studies of inhaled iloprost and studies of digitally monitored daily physical activity in pulmonary arterial hypertension. Studies of inhaled iloprost (with traditional or digital endpoints) are shown above the timeline, and other studies in pulmonary arterial hypertension with digital monitoring of daily physical activity are shown below the timeline. Blue shading indicates studies with digital monitoring of daily physical activity. Published studies are positioned on the timeline by year of publication; unpublished studies are shown with their ClinicalTrials.gov ID numbers and positioned on the timeline by year of (anticipated) study completion. CTEPH: chronic thromboembolic pulmonary hypertension; e-MOTION PH: electronic activity level monitoring pilot in pulmonary hypertension; ERA: endothelin receptor antagonist; LONGACT: correlation of long-term wrist actigraphy recorded physical performance and 6-min walk distance in patients with pulmonary arterial hypertension; mHealth: mobile health intervention in pulmonary arterial hypertension; MOTION: measuring outcomes in patients with pulmonary arterial hypertension not on active treatment; PAH: pulmonary arterial hypertension; PH: pulmonary hypertension; PHANTOM: pulmonary hypertension and anastrozole trial; TRACE: effect of selexipag on daily life physical activity of patients with pulmonary arterial hypertension; VENTASTEP: evaluation of inhaled iloprost effects using the Breelib nebulizer, on clinical outcomes and physical activity of patients with advanced pulmonary arterial hypertension; WHOLEi+12: whole muscle exercise training in pulmonary hypertension; ZHPHCohort: Zürich Pulmonary Hypertension Outcome Assessment Cohort.

An overview of digital studies in PAH and a timeline of digital studies in relation to key iloprost studies are presented in Multimedia Appendix 2 and Figure 3, respectively [2,3,5,7,9-16,20,30-44].

Physical activity is becoming increasingly important in PAH, with evidence emerging that exercise training in patients with PAH leads to substantial improvements in exercise capacity and pulmonary hemodynamics [45,46]. Moreover, as shown in Figure 3, tracking of daily physical activity is gaining increasing acceptance in PAH studies. Nevertheless, it is still underrepresented as a primary endpoint in PAH clinical trials, which may change in future.

The innovative, digital methodology of the VENTASTEP study will allow simultaneous tracking of daily physical activity, heart rate, and iloprost inhalations for the first time and will provide new insights into clinical outcomes, daily physical activity, and HRQoL in patients starting inhaled iloprost in addition to existing PAH therapy. This is a key strength of this study; the simultaneous capturing of inhalation data and daily physical activity data via remote monitoring will avoid the bias associated with self-reporting and will allow objective assessment of disease course and treatment response. Moreover, it will provide insight into the association of heart rate variability with daily physical activity parameters and intake of PAH medication. The study uses a newly designed app, which harnesses technology available in a consumer wearable and smartphone to measure a wide range of digital parameters; the app is able to distinguish between different types of movement, building a realistic picture of daily physical activity in patients with PAH. The duration of the study (3 months±2 weeks) will also provide a rich digital dataset; previously published studies that included digital measurement of daily physical activity in PAH had shorter monitoring periods (≤14 consecutive days; Multimedia Appendix 2).

Furthermore, VENTASTEP includes traditional investigator- and patient-reported outcomes such as 6MWD and HRQoL (measured using the EQ-5D) alongside digital monitoring data, allowing the potential identification of new digital markers of disease progression and treatment response in PAH. The 6MWD is a widely used measure in PAH [17,18], but it reflects maximum physical activity rather than average daily physical activity. HRQoL is known to be impaired in patients with PAH [47], which might be reflected by reduced levels of daily physical activity. Assessment of daily physical activity could thus provide greater insight into the behavior of patients and the impact of PAH in real life and may allow the development of behavioral interventions to improve outcomes.

In our single-center survey of 30 patients with PAH, 50% (15/30) of the patients indicated that they would accept activity tracking. The rate of acceptance of activity tracking was previously reported as 81% (81 of 99 participants) in a US-based study using the Dynamo Activity Tracker [48] but may differ between devices [49]. Acceptance may also decline with increasing duration of use; a study of 1258 health plan members given tracking devices in the United States found that at least 90.22% (1135/1258), 82.51% (1038/1258), and 74.80% (941/1258) of participants used their devices for at least 6, 9, and 12 months, respectively [50], whereas a study of the Fitbit Zip in France (N=711) showed continued tracking in 73.9% (526/711) and 16.0% (114/711) of participants at 100 days and 320 days, respectively [51]. The VENTASTEP study was designed considering the results of our single-center survey and will give insight into the willingness of patients with PAH to participate in a 3-month digital noninterventional study, which will involve following and accepting digital monitoring procedures over the observation period.

### Limitations

An important limitation of this study is the fact that all participants are aware of being monitored. This knowledge may influence the measures being collected (Hawthorne effect). The study is being performed locally in Germany, and the VENTASTEP study population may not be representative of other countries. A population-based study showed that middle-aged German adults have very low levels of daily physical activity [52]; the difference between impaired and *normal* levels of daily physical activity in the VENTASTEP study may therefore be small. In addition, only patients willing to use wearable devices are included; therefore, the study population may not be representative of the German population with PAH. Physicians will be asked to document all patients in a screening log and record the reasons for noninclusion. All patients in this study are participating in a patient support program, which may also limit the representativeness of the results.

The VENTASTEP study is an observational study based on routine clinical practice; documentation of baseline characteristics may therefore lack detail, the duration of the baseline period will vary between patients and may be insufficient in some cases, and the follow-up visit at the end of the observation period may be delayed in some cases. In addition, patients who drop out because of deterioration cannot be included in the primary analysis. However, the potential impact of this will be estimated with a sensitivity analysis. Gaps in the daily physical activity dataset can arise if the patient does not wear the watch (this may be a particular concern for elderly patients), or if the smartphone and wearables do not work because of failure or battery lifetime. However, these states are detected and flagged by the study app and are excluded from the data evaluation.

Algorithms for classification of device data are derived from people without PAH. The categories might not be appropriate for patients with PAH. This is especially true for heart rate assessments related to parameters reflecting daily physical activity. In addition, deviations from normal heart function (eg, valve insufficiencies and pacemakers) might lead to bias. The study app and iPhone and Apple Watch tracking systems have not been tested in patients with PAH previously, and no comparison data with other activity trackers are available.

The design of this nonrandomized, single cohort study will not allow differentiation between effects induced by treatment and the natural course of disease. The study does not directly measure physical fitness, which is a separate concept from daily physical activity [53] although the 2 are related [54].

### Conclusions

The design of the VENTASTEP study represents a substantial advance in the evaluation of digital monitoring of daily physical activity in PAH. The VENTASTEP study includes rigorous analysis of multiple daily physical activity parameters, heart rate, and iloprost inhalation, monitored simultaneously using digital technology in patients with PAH over a substantial period (3 months±2 weeks) in a real-world setting. Changes in traditional clinical measures of disease course and treatment response are also assessed and their correlation with changes in digital measures of daily physical activity and 6MWD will be evaluated as the primary objective. The study will thus provide a wealth of data to inform future research on the utility of digital parameters as outcome assessment tools for disease monitoring and guidance of treatment. The study will also provide insight into clinical outcomes, daily physical activity, and HRQoL in patients starting inhaled iloprost, in addition to existing PAH therapy.

## Appendix

Multimedia Appendix 1 Additional information on data transfer and processing.

Multimedia Appendix 2 Studies of digital monitoring parameters in pulmonary arterial hypertension.

## Abbreviations

6MWD: 6-min walk distance
BNP: brain natriuretic peptide
CRO: contract research organization
EQ-5D: EuroQol 5-dimensions questionnaire
ERA: endothelin receptor antagonist
GPS: global positioning system
HRQoL: health-related quality of life
NT-proBNP: N-terminal pro–brain natriuretic peptide
PAH: pulmonary arterial hypertension
WHO FC: World Health Organization functional class
