# Supplementary material for: Evaluation of Clinical Outcomes and Simultaneous Digital Tracking of Daily Physical Activity, Heart Rate, and Inhalation Behavior in Patients With Pulmonary Arterial Hypertension Treated With Inhaled Iloprost: Protocol for the Observational VENTASTEP Study
Source: JMIR Res Protoc. 2019 Apr 15;8(4):e12144. doi: 10.2196/12144 (PMC6487342; doi:10.2196/12144)
Supplement: Multimedia Appendix 1 [file resprot_v8i4e12144_app1.pdf]

Multimedia Appendix 1

Evaluation of Clinical Outcomes and Simultaneous Digital Tracking of Daily Physical Activity, Heart Rate, and Inhalation Behavior in Patients with Pulmonary Arterial Hypertension Treated with Inhaled Iloprost: Design of the Observational VENTASTEP Study

Christian Mueller, PhD; Barbara Stollfuss, MD, PhD; Alexander Roitenberg, MD; Jonas Harder, MD, PhD; Manuel J Richter, MD

Additional Information on Data Transfer and Processing

If the smartphone is not connected to the wearable, daily physical activity and heart rate data will be stored locally on the wearable and transferred at the next possible time (data saved in the 6-minute walking distance [6MWD] app can be stored in the wearable until the end of the study; other data can be stored in the wearable for up to 72 hours, after which they are overwritten with new data). If a smartphone does not connect with the cloud server for several days, this is detected by xbird. Only xbird has access to the data in the cloud server.

After completion of the study and final analysis by the contract research organization (CRO), all pseudonymized source data will be transferred to Bayer. Both the wearable and the smartphone will be returned by the patients after the end of the observation period. The data will then be deleted from the wearable and smartphone (by Vodafone GmbH), from the cloud server (by xbird), and from the electronic data capture system (by the CRO).
