# Supplementary material for: Evaluation of Clinical Outcomes and Simultaneous Digital Tracking of Daily Physical Activity, Heart Rate, and Inhalation Behavior in Patients With Pulmonary Arterial Hypertension Treated With Inhaled Iloprost: Protocol for the Observational VENTASTEP Study
Source: JMIR Res Protoc. 2019 Apr 15;8(4):e12144. doi: 10.2196/12144 (PMC6487342; doi:10.2196/12144)
Supplement: Multimedia Appendix 2 [file resprot_v8i4e12144_app2.pdf]

Multimedia Appendix

Evaluation of Clinical Outcomes and Simultaneous Digital Tracking of Daily Physical Activity, Heart Rate, and Inhalation Behavior in Patients With Pulmonary Arterial Hypertension Treated With Inhaled Iloprost: Design of the Observational VENTASTEP Study

Christian Mueller, PhD; Barbara Stollfuss, MD, PhD; Alexander Roitenberg, MD; Jonas Harder, MD, PhD; Manuel J Richter, MD

Table. Studies of digital monitoring parameters in PAH.

Study	Indication, study design	Duration of continuous activity tracking	Digital monitoring device(s)	Digital endpoints
Aslan et al. [14]	PAH/CTEPH, obs. study (n = 57)	At least 3 days	SenseWear Pro armband	Total energy expenditure, physical activity duration, active energy expenditure, no. of steps, and average METs
WHOLEi+12 trial [33-35]	PAH/CTEPH, RCT of exercise training vs standard care (n = 40)	5–10 days (including 2 weekend days); minimum 10 h complete data per day; if > 7 days, used only data for last 7 days including weekends	ActiGraph GT3X (worn on waist)	Average weekly time engaged in sedentary behavior, light physical activity and moderate-to-vigorous physical activity
Gonzalez-Saiz et al. [36]	PAH/CTEPH, obs. study (n = 75 patients and n = 107 controls without PH)	5–10 days (including weekends); minimum 10 h complete data per day	ActiGraph GT3X (worn on waist)	Average daily time engaged in inactivity or different intensities of physical activity
Mainguy et al. [12]	iPAH/PAH-SSc, obs. study (n = 50)	1 week	SenseWear Pro armband	Energy expenditure, duration of physical activity above pre-determined intensity level (> 3 METs), and no. of steps

MOTION trial [7][a]	PAH, single-arm, open-label, phase 4 trial of riociguat (n = 75)	During 6MWD tests	NS	Accelerator band activity recorded during 6MWD test at screening, day 0, week 16 and week 24
Matura et al. [9]	PAH, obs. study (n = 15)	1 week	ActiGraph wGT3X-BT (worn on hip)	Average intensity and standard deviation of the composite vector magnitude, and episodes of continuous activity with vector magnitude > 40 (90th percentile)
Okumus et al. [10]	PAH/CTEPH, obs. study (n = 45)	At least 72 h	SenseWear armband	Energy expenditure, no. of steps, average METs, active energy expenditure, and sedentary, moderate and vigorous activity duration
Panagiotou et al. [15]	PAH/CTEPH, obs. study (n = 20)	7 days	DynaPort (worn over L2 vertebra)	Daily walking intensity, daily steps, and walking time
Pugh et al. [11]	PAH, obs. study (n = 50)	7 days	ActiGraph GT3X (worn on waist)	Total daily activity counts, sedentary time, and proportion of time at various activity levels
Ulrich et al. [16]	PAH/CTEPH, obs. study (n = 23)	2 weeks	Actiwatch Model AW4 (worn on wrist)	Sleep measures, daytime activity duration, total daytime activity count, and average daytime activity count

Zijlstra et al. [13]	Pediatric PAH, obs. study (n = 89)	7 days	ActiGraph wGT3X (worn on hip)	Vector magnitude counts per minute, and time per day spent in different physical activity intensity levels
NCT02371733 - Aerobic Exercise Training in PAH [37][b]	PAH, RCT of upper extremity aerobic exercise training vs alternative upper extremity exercises (n = 30)	6 weeks	NS	Physical activity (activity monitor)
NCT03078907 – TRACE [39][b]	PAH, RCT of selexipag vs placebo (n = 100)	2 weeks	NS (wrist actigraphy)	Change from baseline to week 24 in daily time spent in non-sedentary activity, % daily time spent in non-sedentary activity, total daily life physical activity, total sleep time, wake after sleep onset, no. of awakenings, and sleep efficiency
NCT02309463 - LONGACT[c] [44]	PAH, obs. study of patients newly initiating ERA therapy	54 weeks	NS (wrist actigraphy)	Activity score and change of physical activity

NCT03069716 – mHealth [40][b]	PAH, RCT of a mobile health intervention vs standard care (n = 50)	17 weeks (2 weeks run-in, 12 weeks intervention vs standard of care, 3 weeks follow-up)	Fitbit Charge HR (worn on wrist)	Daily step count, daily goal attainment, daily aerobic time, fidelity of data, and resting heart rate
NCT03229499 – PHANTOM [41][b]	PAH, RCT of anastrozole vs placebo (n = 84)	Unclear (maximum 12 months)	NS (actigraphy)	Change in actigraphy-measured physical activity at 3, 6, and 12 months
NCT02909608 - Actigraphy in Pediatric PH [38][b]	Pediatric PAH, obs. study (n = 200)	2 weeks	NS (actigraphy)	Utility of the measurement properties of actigraphy, and correlations of actigraphy with disease severity, progression, clinical worsening, and survival
NCT02536534 - e-MOTION PH [32][d]	PAH/CTEPH, obs. study (n = 35)	6 months	Fitbit Flex (worn on wrist)	No. of steps per day at baseline, 3 months, and 6 months
NCT02558582 - Effect of Exercise Training in Patients With PH [42][b]	PH, RCT of respiratory exercise therapy ± supplemental oxygen (n = 32)	Unclear (maximum 12 months)	NS (actigraphy)	Energy expenditure, steps per day, sleep time and efficiency, lying down time, physical activity level, and METs at baseline, 3 weeks, 15 weeks, 6 months, and 12 months
NCT02249806 – ZHPHCohort [43][b]	Precapillary PH, obs. study (n = 100)	10 years	NS (actigraphy)	Motion, step counts, and METs at 3, 6, and 12 months and yearly thereafter

NCT03293407 – VENTASTEP[b]	PAH, obs. study of patients receiving inhaled iloprost via the Breelib™ nebulizer (n = 80)	3 months	Apple Watch 2 (worn on wrist) and iPhone with xbird study app; Breelib™ nebulizer with BreeConnect™ app	Daily physical activity (distance walked, no. of steps, no. of floors climbed, time spent at home, no. of relevant location changes, no. of times leaving home, and no. of times standing up), 6MWD, heart rate, and iloprost inhalation frequency, completeness, and duration

[a]Trial design paper published in 2017; study completed according to ClinicalTrials.gov record but results not yet published (checked 24 July 2018).

[b]Study recruiting according to ClinicalTrials.gov record.

[c]This study was withdrawn (last ClinicalTrials.gov update posted in 2016); the reported reason for the withdrawal was because the EC did not accept measurement of patient activity for the primary end-point with a non-validated activity tracking device.

[d]Study completed according to ClinicalTrials.gov record but not yet published.

6MWD, 6-minute walking distance; CTEPH, chronic thromboembolic pulmonary hypertension; e-MOTION PH, Electronic Activity Level Monitoring Pilot in Pulmonary Hypertension; ERA, endothelin receptor antagonist; iPAH, idiopathic pulmonary arterial hypertension; LONGACT, Correlation of Longterm Wrist Actigraphy Recorded Physical Performance & 6-minute Walk Distance in Patients With Pulmonary Arterial Hypertension; MET, metabolic equivalent; mHealth, A Mobile Health Intervention in Pulmonary Arterial Hypertension; MOTION, Measuring Outcomes In Patients With Pulmonary Arterial Hypertension Not on Active Treatment; no., number; NS, not specified; obs., observational; PAH, pulmonary arterial hypertension; PH, pulmonary hypertension; PHANTOM, Pulmonary Hypertension and Anastrozole Trial; RCT, randomized controlled trial; SSc, systemic sclerosis; TRACE, Effect of Selexipag on Daily Life Physical Activity of Patients With Pulmonary Arterial Hypertension; VENTASTEP, Evaluation of Inhaled Iloprost Effects Using the Breelib™ Nebulizer, on Clinical Outcomes and Physical Activity of Patients With Advanced Pulmonary Arterial Hypertension; WHOLEi+12, Whole Muscle Exercise Training in Pulmonary Hypertension; ZHPHCohort, Zürich Pulmonary Hypertension Outcome Assessment Cohort.

References

Please see bibliography in main manuscript.
